# Formation and dissolution of microbubbles on highly-ordered plasmonic nanopillar arrays

**DOI:** 10.1038/srep18515

**Published:** 2015-12-21

**Authors:** Xiumei Liu, Lei Bao, Michele Dipalo, Francesco De Angelis, Xuehua Zhang

**Affiliations:** 1 School of Mechatronic Engineering, China University of Mining and Technology, Xuzhou, 221116, China; 2 Physics of Fluids Group, Faculty of Science and Technology, University of Twente, Enschede, 7500 AE, The Netherlands; 3 Softer Matter and Interfaces Group, School of Civil, Environmental and Chemical Engineering, RMIT University, Melbourne, VIC 3001, Australia; 4 Istituto Italiano di Tecnologia, Genova 16163, Italy

## Abstract

Bubble formation from plasmonic heating of nanostructures is of great interest in many applications. In this work, we study experimentally the intrinsic effects of the number of three-dimensional plasmonic nanostructures on the dynamics of microbubbles, largely decoupled from the effects of dissolved air. The formation and dissolution of microbubbles is observed on exciting groups of 1, 4, and 9 nanopillars. Our results show that the power threshold for the bubble formation depends on the number density of the nanopillars in highly-ordered arrays. In the degassed water, both the growth rate and the maximal radius of the plasmonic microbubbles increase with an increase of the illuminated pillar number, due to the heat balance between the heat loss across the bubble and the collective heating generated from the nanopillars. Interestingly, our results show that the bubble dissolution is affected by the spatial arrangement of the underlying nanopillars, due to the pinning effect on the bubble boundary. The bubbles on nanopillar arrays dissolve in a jumping mode with step-wise features on the dissolution curves, prior to a smooth dissolution phase for the bubble pinned by a single pillar. The insight from this work may facilitate the design of nanostructures for efficient energy conversion.

Noble metal nanostructures illuminated at a wavelength coinciding with their plasmon resonance have been demonstrated to generate photo-induced heating with high efficiency^1^,^2^,^3^. This localized plasmonic heat can be sufficiently high to vaporize the liquid adjacent to nanostructures, or to drive the local catalytic reactions. Such a process has significant potential for various applications^4^,^5^,^6^,^7^,^8^,^9^,^10^. For instance, microbubbles may act as effective lenses in guiding the propagation of surface waves^11^, or as micropumps for liquid transport and mixing in a microfluidic environment^12^. Plasmonic microbubbles are also powerful tools for probing and manipulating biological systems^9^,^13^, photothermal imaging^14^, drug and gene delivery, photoacoustic imaging^15^,^16^,^17^, and induced membrane rupture and death of targeted cells in therapeutics^18^,^19^,^20^,^21^,^22^,^23^,^24^,^25^. In plasmonic-assisted catalytic reactions, the bubble shells that wrap around the plasmonic nanoparticles may lead to local sharp temperature rise and trigger exothermic chemical reactions^26^. It is also reported that in water desalination by plasmonic heating, bubbles may carry those suspended nanoparticles to the liquid surface, playing an important role in the vapor release^27^. In many processes mentioned above, it is unavoidable that plasmonic nanobubbles develop to microbubbles before they are released from the system. Although intensive research interest has been drawn on nanobubble dynamics^2^,^10^, the knowledge on the entire process of the plasmonic bubble evolution, especially on the microscale remains largely unexplored.

Recent studies showed that dissolved air in liquid is crucial for the plasmonic bubble nucleation and long lifetime^4^. Adleman *et al.*^28^ suggested that the dissolved gas in liquid facilitates the vapor bubble formation, according to the bubble behaviors in miniaturized heterogeneous catalytic reactions. Baffou *et al.*^4^ found that the lifetime of these bubbles is as long as several minutes, which is attributed to the key role of the dissolved gas for the growth and stability of the light-induced microbubbles. Namely, the bubbles consist of not only water steam but also air dissolved in the liquid. Otherwise, one would expect the emerging vapor bubble to collapse and condense immediately once it gets into contact with the cold liquid^4^.

In addition to the influence of dissolved air, the dynamics of plasmonic-induced microbubbles depends on the properties of the substrate on which the plasmonic structures reside. For instance, the wettability and heterogeneities on the supporting substrate may influence the shape and the expansion and dissolution of the bubble.

In this work, we investigate in the intrinsic effects of the number of plasmonic nanostructures on the formation, growth and dissolution of microbubbles, largely decoupled from the effects of dissolved air. The plasmonic nanostructures in our study are three-dimensional vertical nanopillars arranged in highly ordered arrays. These nanopillars present many advantages in respect to more conventional 2D nanostructures. Firstly, thanks to their high cross section and high absorbance resulting from their out-of-plane configuration, the nanopillars possess high harvesting capabilities and high field enhancements, compared to their planar counterparts with the resonant frequency in the visible range^26^. Secondly, the tips of nanopillars are away from the supporting substrate; therefore, the heat transfer from the nanopillars to the substrate is minimized under the experimental conditions. Thirdly, the vertical nanopillars lift and pin the bubbles during expansion and dissolution, thus the bubble dynamics is not influenced by any random heterogeneities on the substrate. Furthermore, in a regular array, the number of excited plasmonic nanopillars contributing to the bubble formation is determined by the inter-pillar distance that is controlled in nanofabrication precisely. In addition, the number of pillars supporting a bubble with a certain size can be estimated based on the pillar spacing.

By using degassed water to alleviate the influence of dissolved air on the plasmonic bubble formation, we study how the heating threshold for bubble nucleation and the maximal bubble size vary when the plasmonic heating is generated by groups of 1, 4 or 9 nanopillars. We show that the pinning effect from the underlying pillars leads to the jumping mode of the bubble dissolution. Our study reveals that the spatial arrangement of nanopillars has significant effects on the entire evolution of the plasmonic bubble including the formation, growth and dissolution. Less densely arranged nanopillars show a reduced pinning of dissolving bubbles and result in a faster release of bubbles from the nanopillars.

## Materials and Methods

### Sample Description

**Figure 1:**
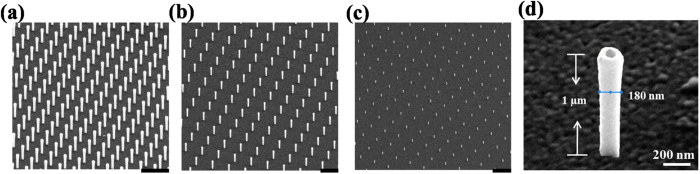
SEM images of gold nanopillar arrays with (a) interspace of 1 μm, (**b**) 2 μm and (**c**) 4 μm. The length of scale bar in (**a,b**) is 2 μm and in (**c**) is 5 μm. (**d**) SEM image of a representative gold nanopillar.

The patterns consist of regular arrays of vertical cylindrical gold nanopillars on a flat quartz coverslip (Fig. 1(a–c)). These nanostructures are obtained with a novel Focus Ion Beam based technique reported in a previous work^29^. The nanopillars have a hollow geometry as shown in Fig. 1(d) and possess high broadband plasmonic field enhancement^30^,^31^. More specifically, the nanopillars consist of a polymeric hollow nanocylinder with internal and external diameters of respectively ~100 nm and ~130 nm; on the external nanocylinder wall a 25 nm thick gold layer is deposited, resulting in a total external diameter of about 180 nm. The nanocylinders are produced, starting with the polymer spin-coated on the quartz coverslip. The final height of the nanocylinders is defined by the thickness of this polymer film and is therefore very homogeneous among the nanostructures. The thickness can be tuned precisely by changing the spinning conditions.

The pitches of nanopillar arrays vary from 1 μm to 4 μm (Fig. 1(a–c)). Each array contains 20 × 20 gold nanopillars and the distance between the arrays is 500 μm. The gold coating is about 25 nm on the nanopillars and about 15 nm on the quartz substrate^26^. In the experiments on the hydrophobic substrate, the entire substrate was further coated with a layer of ~10 nm Teflon. The transmission spectra of nanopillar arrays with and without Teflon coating were measured using a white light source from Spectral Products and a spectrometer USB4000 from Ocean Optics. The light was directed on the sample through an optical fiber and collected with a 20× optical objective. An aperture of 50 μm in diameter was used to define the light spot. Large nanopillars arrays over 60 μm × 60 μm were fabricated on quartz in order to cover the light spot completely. The spectra were referenced to the absorption properties of the thin flat gold layer on the substrate in the vicinity of the nanopillars arrays.

Both air-equilibrated water and degassed water were used in the experiments. To prepare degassed water, a bottle of water (Milli-Q, 18.3 MΩ) was put into a sealed round flask connected to an external pump. The water was stirred as the flask was pumped vacuum, and the degassing process was completed after one hour when no more bubbles were visible. The treated water was used in the experiment immediately. Strictly speaking, the degassed water was turned to partially degassed water during the experiments because it was unavoidable that some air re-dissolved into water.

### Set-up for the microbubble formation

**Figure 2:**
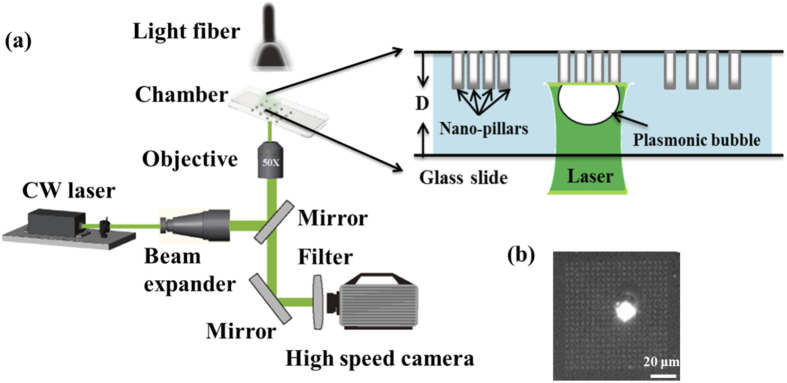
(**a**) Schematic of the experimental setup. The laser was illuminated on the hanging nanopillar array. The top-view of microbubble was monitored from the bottom of the fluid chamber. (**b**) A representative image of the microbubble generated by plamonsic heating on the nanopillar arrays. The length of scale bar is 20 μm.

The experimental setup to monitor the formation, growth and dissolution dynamics of plasmonic microbubbles is shown in Fig. 2(a). A CW laser (Cobolt Samba^TM^) with 532 nm wavelength was used to heat the nanopillar arrays. The laser beam went through a beam expander (10×). The power of CW laser illumination was controlled by a variable attenuator (Zaber technologies T-RS60A). In the experiments, the laser power was tuned up gradually till the onset of a bubble formation and the critical laser power was marked.

The laser was focused with a microscope objective (Olympus 50×, 0.45 NA) and hit the hanging nanopillars on the top wall of the fluid chamber shown in Fig. 2(b). The chamber was filled with water, and the gap between top and bottom glass slides was 1 mm. The top-view of the plasmonic bubble was recorded by a high-speed camera with a frame rate of 500 fps (Photron Fastcam SA2). The illumination for the fast camera was provided by the filtered light from a fiber lamp (Olympus ILP-1). The spatial resolution limit was ~0.75 μm.

## Results and Discussion

### Coupled effects from dissolved air and nanopillar number on plasmonic bubble formation

**Figure 3:**
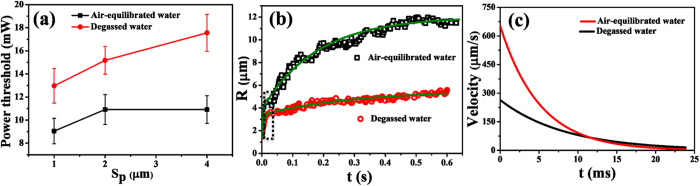
Bubble growth on nanopillar arrays with different inter-distance between each nanopillars (*S*_*p*_) in air-equilibrated water and degassed water. (**a**) Threshold of laser power for the plasmonic bubble formation in air-equilibrated water and in the degassed water. Squares represents the situations in air-equilibrated water and the circles are in the degassed water. The standard deviation of power threshold values was ca. 1.30 mW for four independent measurements. In air-equilibrated water and the degassed water under the same illumination, the bubble radius in (**b**) and the growth velocity of the bubble radius in (**c**) are plotted as a function of time. The inter-pillar spacing is 1 μm in both cases. The standard deviation of measured radius values was ca. 0.3 μm for three independent measurements.

We first examined the laser power threshold for the bubble formation on the nanopillar arrays with the interspacing of 1 μm, 2 μm and 4 μm. The plot in Fig. 3(a) shows the critical power as a function of the pillar spacing in air-equilibrated and the degassed water. If the power is lower than the critical power, no matter how long the exposure time of the laser, no microbubble was observed. As expected, the power threshold on the same pitch of nanopillars is always higher in degassed water. The difference between the critical powers of air-equilibrated and degassed water scales with the nanopillars spacing; it is approximately 30%, 40% and 60% higher in degassed water than in air-equilibrated water at the nanopillars spacing of 1 μm, 2 μm and 4 μm, respectively. Clearly, the dissolved air in the liquid facilitates the bubble nucleation, particularly when lower overheating was generated on sparsely spaced plasmonic nanopillars.

The plot in Fig. 3(a) shows that the power threshold is 13 mW on the 1 μm array, but increases to 17.5 mW on the 4 μm array in degassed water. In contrast, it changes only slightly from 10 mW to 11 mW on the same nanopillar arrays in air-equilibrated water. The power threshold for the bubble formation on different number density of nanopillars is notably less pronounced in the presence of dissolved air in the water, indicating that the intrinsic effect of the nanopillar number on the bubble formation is coupled with the effect of dissolved air.

Under the same incident laser power (~12.97 mW), we followed the bubble radius (*R*) on 1 μm-spaced nanopillars and plotted the bubble radius as a function of time in Fig. 3(b). For a quantitative analysis, the expansion velocity of bubble was derived from differentiating the fitting curve in Fig. 3(c). The maximal initial grow velocity of the plasmonic bubble in air-equilibrated water reached 650.3 μm/s, which is much faster than the bubble expansion velocity of 263.8 μm/s in the degassed water.

This clearly shows that besides the power threshold for the bubble nucleation, the presence of the dissolved air in water has significant influence on the growth rate and the maximal size of plasmonic bubbles. Hence, it is essential that the dissolved air must be largely removed to decouple the contribution of the nanopillar properties from that of the dissolved air. In the following section we investigate the effects from nanopillar spacing under the conditions of degassed water.

### Nanopillar spacing and maximal bubble size

**Figure 4:**
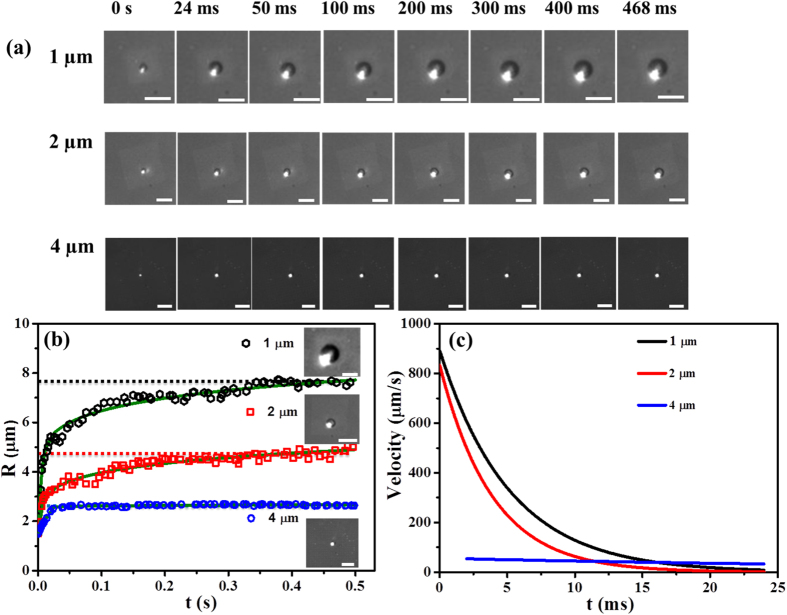
Growth process of bubbles on nanopillar arrays with different inter-pillar spacing in the degassed water. (**a**) The optical images show the growing microbubbles under given conditions. The length of scale bar is 20 μm. The plot in (**b**) shows the bubble radius as a function of time. The inserted images correspond with the maximal bubble size on the nanopillar arrays with inter-spacing of 1 μm (top), 2 μm (middle) and 4 μm (bottom) for 0.5 s. Length of scale bars: 10 μm (top image); 20 μm (middle and bottom images). The plot in (**c**) shows the bubble radius growth velocity as the function of time. We set *t* = 0 as bubbles grew to 1.5 μm in each case.

Fig. 4(a) shows the growth of a plasmonic microbubble with illumination time on nanopillar arrays with different spacing in the degassed water. The bubble radius as a function of illumination time on nanopillar arrays has been plotted in Fig. 4(b). The plot in Fig. 4(b) shows the bubble growth velocity as the function of illumination time. By the end of the fast growth, the size of the bubbles is approximately 2 μm, 3.1 μm and 5.5 μm for the interspacing of 4 μm, 2 μm and 1 μm, respectively. After 0.5 s, the bubble growth approaches a steady state, with the maximal bubble size being 2.2 μm, 4.5 μm and 7.8 μm for the above mentioned inter-spacings. Clearly, the maximal radius of a plasmonic bubble was larger for more densely packed pillars.

Below we show that at the final steady state, the generated heat from the nanopillars is balanced by the energy loss from the bubble to the surrounding liquid at room temperature. The temperature distribution in arrays of plasmonic sphere nanoparticles on substrate has been investigated in previous works^32^,^33^. According to Fourier’s law, the heat flux out of a spherical bubble with a radius of *R* and temperature gradient (*∂*_*r*_*T*) at edge is


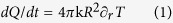


where *Q* is heat power (J), *k* is thermal conductivity of the material (J s^-1^m^-1^K^-1^). With the assumption *∂*_*r*_*T *≈ Δ*T/R*,


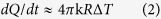


where Δ*T* is temperature difference across the bubble surface (K). The temperature difference (Δ*T*) is assumed to be the same for all the bubbles, so at the steady state the heat loss from the bubble is proportional to the bubble radius (*R*). This relationship holds for the mass transfer for evaporating droplets or dissolving bubbles, which have been extensively studied in literature^34^.

**Figure 5:**
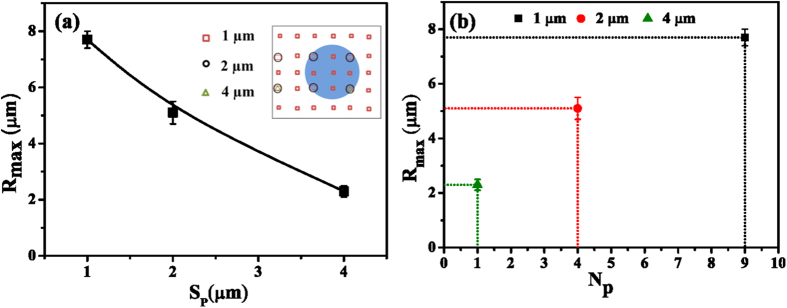
Bubble maximal radius on the nanopillar arrays. (**a**) Maximal bubble radius (*R*_max_) versus the nanopillar spacing (*S*_*p*_). The inserted sketch illustrates the number of nanopillars with spacing of 1 μm, 2 μm and 4 μm, respectively, covered by the 2.9 μm laser spot. (**b**) The plot of the maximal bubble radius (*R*_max_) versus the number of nanopillars (*N*_*p*_) under illumination. The deviation was obtained from three independent measurements.

Thanks to the highly regular arrangement of the nanopillars, we can further estimate the total heating efficiency of a single nanopillar from the maximal bubble size. The illumination area was determined by the laser diameter that was constant in all experiments. The number of excited nanopillars under illumination is calculated from the nanopillar spacing and from the laser diameter of 2.9 μm, considering the assumption that those nanopillars outside of the bubble footprint did not contribute to the heat generation. Within the circular area of illumination in the insert of Fig. 5(a), there were 9 pillars for the 1 μm arrays, 4 pillars for the 2 μm arrays and 1 pillar for the 4 μm arrays. We also know from the experiments that the bubble on the 4 μm array is produced by heating a single nanopillar, as the laser had to be aligned to certain locations before the bubble generation. The maximal bubble radius versus the inter-spacing of nanopillars and versus the number of illuminated pillars is plotted in Fig. 5(a,b), respectively. The maximal bubble radius is approximately proportional to the number of illuminated nanopillars, suggesting that the heat generated from single pillars may be added up to the entire heat required to maintain the maximal bubble size at the steady state.

### Surface wettability of the nanopillars

**Figure 6:**
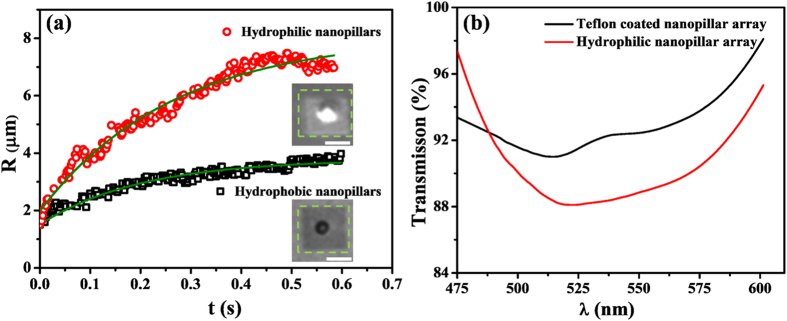
(**a**) Bubble radius as function of time on the substrate with and without coating. The inter-pillar spacing is 1 μm and the laser intensity is the same. The standard deviation of measured radius values was ca. 0.3 μm for three independent measurements. The inserted images are representative optical images of a bubble grown on hydrophilic nanopillar arrays (top) and on hydrophobic nanopillar arrays (bottom) at 0.5 s. The scar bar: 10 μm. (**b**) The transmission spectra of nanopillar array without Teflon coating and with Teflon coating.

We examined bubble growth behavior on nanopillars arrays that were hydrophobized with a 10 nm thick layer of Teflon coating. As a first experiment, we compare the power threshold on the pillars with and without the coating in the air-equilibrated water. The laser power is 9.08 mW on the hydrophilic sample and 10.92 mW on the hydrophobic sample with inter-spacing of 1 μm. Therefore, the threshold is higher on the Teflon coated nanopillars, suggesting that the hydrophobic coating reduces the light-heat conversion efficiency of the nanopillars. The plot in Fig. 6(a) shows the bubble radius as a function of time. The overall features of the bubble growth on both nanopillar arrays are similar; however, the maximal bubble radius was smaller on coated nanopillars under the same illumination. As shown in Fig. 6(b), on the hydrophilic nanopillar arrays, a broad absorption band appears in the range of 475 nm to 600 nm with the maximum absorption at ~520 nm. This wavelength is close to the laser wavelength 532 nm used in experiment. This suggests that after Telfon coating, the absorption spectra of nanopillar array is comparable to that of hydrophilic nanopillar arrays. Therefore, we attribute a smaller bubble on the hydrophobic substrate than on the hydrophilic substrate to the reduced conversion efficiency to heat^3^. We also noticed that the bubbles on hydrophobized nanopillars are not as reflective as those on the uncoated array, suggesting that bubbles are flatter due to a higher contact angle of water (i.e. a smaller angle for the bubble) on the hydrophobic substrate.

**Figure 7:**
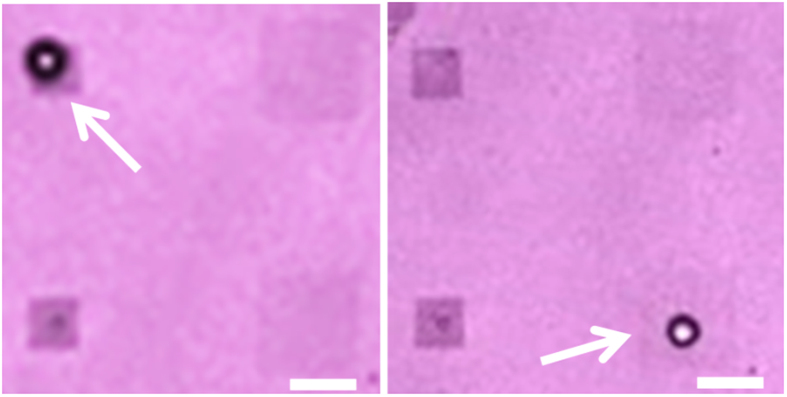
Optical images of air pockets left on hydrophobic nanopillar arrays after bubble dissolution. The length of the scale bar is 20 μm.

An interesting observation is that an air pocket remained after the bubble dissolution on the hydrophobic pillar array, as shown in Fig. 7. Those gas pockets regrew under the illumination of the laser below the critical power intensity, suggesting that pre-existing nanobubbles at the interface can expand by plasmonic heating^31^. This result may be of interest in mediating the onset of phase transition by plasmonic heating. The formation of microbubbles by hydrophobic nanopillars could be also integrated on already super-hydrophobic surfaces such as those introduced by Miele *et al.*^35^.

### Dissolution of bubbles on the nanopillar arrays

**Figure 8:**
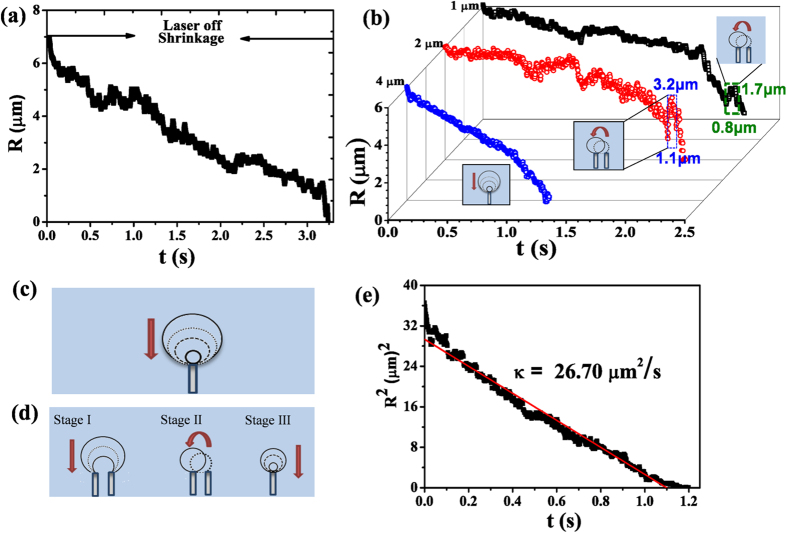
Dissolution of microbubbles on nanopillar arrays. (**a**) The plot of the bubble radius as a function of time. (**b**) Dissolution curves of bubbles on the nanopillar arrays with the interspacing of 4 μm, 2 μm, and 1 μm. (**c**) Sketch of the dissolution of a bubble pinned on a single pillar, corresponding to the blue dissolution curve in (**b**). (**d**) Sketch of the dissolution process of a bubble pinned by multiple pillars. The detachment from the outer pillars leads to the step-wise features on the dissolution curve. (**e**) A plot of the square bubble radius as a function of time for a single bubble dissolved on one nanopillar.

Figure 8(a) presents the dissolution curve of the bubble after the laser was turned off. It takes about 3.5 s till the bubble completely dissolved. The fact that the plasmonic bubble does not collapse as soon as the heating beam is switched off is consistent with the previous report that the bubble is not entirely made of vapor but contains also some air^4^.

Interestingly, we were able to observe a jumping-like mode of dissolving microbubbles. The dissolution curve of a large bubble possesses step-wise features on the arrays with spacing of 2 μm and 1 μm shown in Fig. 8(b). The jumps in the dissolution curves are attributed to the detachment of the bubble boundary from the underlying nanopillars sketched in the inserts in Fig. 8(b–d). When the initial bubble footprint is large enough to span over multiple pillars, the bubble radius jumps up as the bubble boundary depins from an outer pillar. Meanwhile, the contact angle of the bubble increases on the new location while the bubble volume is conserved. Based on the bubble radius, the jumping distance of the contact line across the substrate is around 1 μm, half of the inter-pillar spacing of 2 μm, suggesting the boundary moves along the axis direction or diagonally.

Such jumps in dissolving bubbles are more likely to be observed when the bubble volume is small, a situation similar to the jumps during dissolving microdroplets on a heterogeneous substrate^36^. In the analogous scenario of the droplet spreading on regularly patterned surfaces of square arrays of circular posts, the droplets take various shapes, depending on the liquid contact angle and the topographic features^37^.

It is also remarkable that we were able to capture the dissolution of a bubble pinned by a single nanopillar. This is an unavoidable dissolution phase as the footprint radius of the bubble becomes too small to span over two nanopillars, which was ~0.8 μm and 1.1 μm on the array with spacing of 1 μm and 2 μm (Fig. 8(b)). We note that, thanks to the vertical configuration of the pillars, the pinning effect from the pillars is much stronger than that from any arbitrary defects from the substrate. This is the reason why the dissolution curve of the bubble attached to a pillar is smooth till the bubble vanishes. Specifically, the dissolution curve was smooth all the time for the bubble with an initial radius of ~6 μm on the array with spacing of 4 μm. The plot in Fig. 8(e) shows that the square of bubble radius decreases linearly with the dissolution time, same as a free dissolving bubble in the bulk liquid analyzed by Epstein and Plesset^38^. The consequence of the pinning from the nanopillars is significant for the bubble lifetime. A bubble of a given size dissolved fastest on 4 μm nanopillar arrays and slowest on 1 μm nanopillar arrays.

## Conclusions

In summary, we demonstrate that in degassed water, the plasmonic nanopillar number and the surface hydrophobic coating have significant influence on the entire evolution of plasmonic microbubbles. The overheating threshold and the maximal bubble size are correlated with the pillar number. Specifically, a steady bubble size is approximately proportional to the number of illuminated nanopillars. This is attributed to the balance between the heat loss to the environment and the heating from the total number of illuminated nanopillars. As the microbubbles dissolve, the dissolution phase shows a step-wise behavior due to depinning of the bubble boundary from the underlying nanopillars, prior to the final smooth dissolution phase on a single pillar. The bubble dissolves fastest on the array with the largest inter-pillar distance. Given the significant potential of plasmonic effects in the light-heat conversion, the insights from this work may provide some valuable guidelines for the design of nanostructures to achieve highly efficient bubble formation and release.

## Additional Information

**How to cite this article**: Liu, X. *et al.* Formation and dissolution of microbubbles on highly-ordered plasmonic nanopillar arrays. *Sci. Rep.* **5**, 18515; doi: 10.1038/srep18515 (2015).
